# Fluoroscopic balloon dilation is effective for anastomotic leakage caused by compression due to intraperitoneal hematoma after ileostomy closure: A case report

**DOI:** 10.1097/MD.0000000000041240

**Published:** 2025-01-03

**Authors:** Teppei Kamada, Hironori Ohdaira, Takashi Aida, Daisuke Yamagishi, Junji Takahashi, Keigo Nakashima, Eisaku Ito, Norihiko Suzuki, Taigo Hata, Masashi Yoshida, Eigoro Yamanouchi, Yutaka Suzuki

**Affiliations:** a Department of Surgery, International University of Health and Welfare Hospital, Nasushiobara, Tochigi, Japan; b Department of Radiology, International University of Health and Welfare Hospital, Nasushiobara, Tochigi, Japan.

**Keywords:** anastomotic leakage, fluoroscopic balloon dilation, ileostomy closure, intraoperative hematoma, postoperative bleeding

## Abstract

**Rationale:**

There have been no reports of delayed anastomotic leakage of the ileum caused by compression of an intra-abdominal hematoma due to postoperative bleeding after ileostomy closure.

**Patient concerns:**

We report the case of a 44-year-old man with delayed anastomotic leakage after ileostomy closure caused by the compression of an intra-abdominal hematoma that healed quickly after fluoroscopic balloon dilatation of the bowel stenosis area.

**Diagnoses:**

Postoperative bleeding and hemorrhagic shock occurred on postoperative day 1 after ileostomy closure. Vital signs stabilized after blood transfusion, and computerized tomography-guided drainage was successfully performed to reduce the hematoma volume. However, residual hematoma compressed the ileum on the anal side of the anastomosis, leading to delayed anastomotic leakage.

**Interventions/outcomes:**

Fluoroscopic balloon dilation of the stenotic area through the drain was successfully performed, and the anastomotic leakage healed quickly.

**Lessons:**

In cases of small intestinal anastomotic leakage after postoperative bleeding, compression of the intestinal tract due to a hematoma should be considered. Fluoroscopic balloon dilation through a drain is an effective procedure.

## 1. Introduction

Diverting loop ileostomy during high-risk rectal anastomosis in colorectal surgery helps decrease anastomotic leakage.^[1]^ Nevertheless, the complications associated with ileostomy closure include bleeding, wound infection, bowel obstruction, anastomotic leakage or stenosis, and abdominal wall hernia.^[2]^

In particular, anastomotic leakage after ileostomy closure is a severe complication and often requires extensive small bowel resection or recreate ileostomy during reoperation, which not only reduces the patient’s quality of life but is also sometimes a critical complication.^[3]^ Risk factors for anastomotic leakage after ileostomy closure include low albumin levels, longer intervals to ileostomy closure, diabetes mellitus, and hand-sewn anastomosis have been reported.^[4]^ However, there have been no reports of delayed anastomotic leakage of the ileum caused by compression of an intra-abdominal hematoma due to postoperative bleeding.

Herein, we report a case of anastomotic leakage after ileostomy closure caused by compression of an intra-abdominal hematoma. The anastomotic leakage quickly healed after fluoroscopic balloon dilatation of the stenosis area.

## 2. Patient information

A 44-year-old man was admitted to our hospital with chief complaints of pneumaturia and repeated cystitis. The patient also had a history of hypothyroidism.

Enhanced contrast-enhanced computed tomography revealed multiple diverticula of the sigmoid colon, and the boundary between the diverticulum and the bladder was unclear. Lower gastrointestinal endoscopy revealed a fistula in the rectosigmoid colon approximately 15 cm from the anal verge. Cystoscopy revealed a fistula in the left posterior wall of the bladder. A sigmoidovesical fistula was diagnosed and laparoscopic surgery was planned. Intraoperative findings revealed severe inflammation around the sigmoidovesical fistula, and severe adhesions had also formed between the ileum and bladder. The ileum and bladder were difficult to exfoliate because of severe adhesions. Therefore, we decided to perform a combined bowel resection. The rectosigmoid colon, including the fistula, was mobilized and resected. The bladder fistula was transected and closed using a knotless barbed suture (V-Loc^TM^ 180, Covidien, Mansfield). The sigmoid-rectal anastomosis was reconstructed using the double-stapling technique. The partial ileum resection was reconstructed with functional end-to-end anastomosis (FEEA). In addition to the presence of a 2-site anastomosis, there was severe inflammation in the surrounding area, and considering the risk of anastomotic leakage, a diverting loop ileostomy 30 cm proximal to the ileo-ileal anastomosis was created.

Surgical procedures included laparoscopic high anterior resection, partial small bowel resection, partial cystectomy, and diverting loop ileostomy. The operation time was 236 minutes, and blood loss was 50 mL. The postoperative course was uneventful, and ileostomy closure was planned 1 month after the initial surgery. Under laparoscopic assistance, adhesions between the omentum and abdominal wall around the stoma were removed. Full mobilization was performed around the stoma, and the intestine was pulled outward. The ileum was reconstructed using FEEA. The surgical procedure involved laparoscopy-assisted ileostomy closure. The operation time was 100 minutes, and the blood loss was 30 mL.

## 3. Clinical findings

On the day after surgery, symptomatic shock occurred due to low blood pressure (<80 mm Hg) and tachycardia. Blood tests showed hemoglobin decreased from 15.3 before surgery to 8.6 g/dL after the surgery.

## 4. Diagnostic assessment

**Figure 1.**
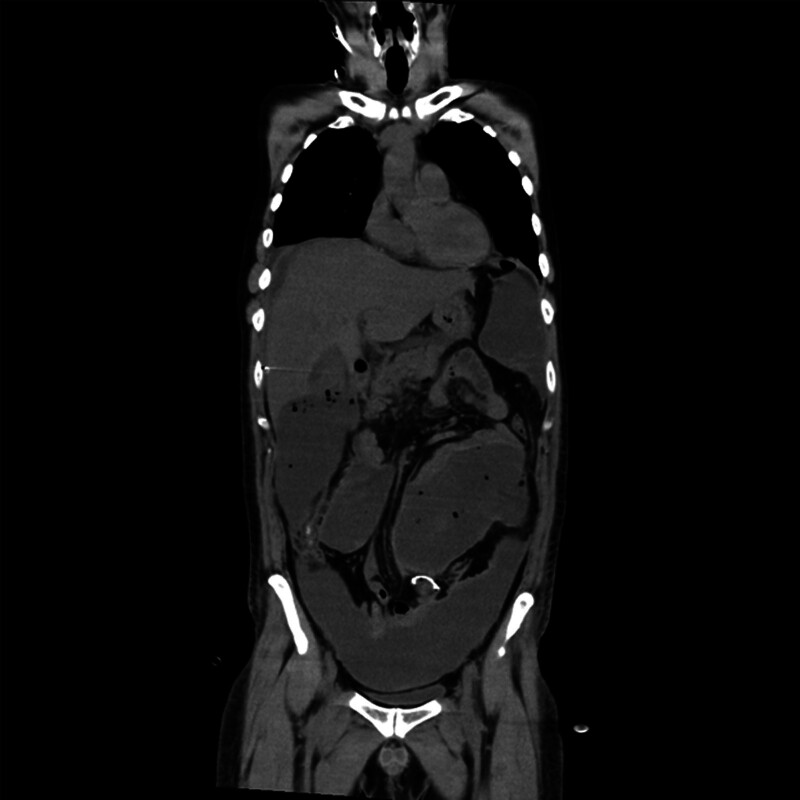
Abdominal computerized tomography on the 8th postoperative day showed extensive intra-abdominal hematoma.

Abdominal computerized tomography (CT) revealed a large amount of high-density fluid in the abdominal cavity (Fig. 1). The diagnosis was postoperative bleeding and hemorrhagic shock. A central venous catheter was inserted, and 8 units of red blood cells and 8 units of fresh frozen plasma were administered to stabilize the vital signs. The vitals stabilized, and no progression of anemia was observed after that. Although hemostasis was achieved, severe abdominal distention and high inflammatory response levels (C-reactive protein [CRP] at 42.9 mg/dL and white blood cell count [WBC] of 18,500/μL) persisted 7 days after surgery.

**Figure 2.**
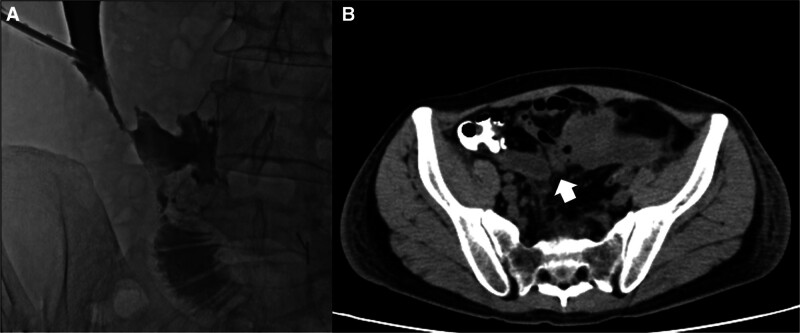
(A) Drain angiography identified the abscess cavity and anastomotic leakage of the ileo-ileal anastomosis. (B) Abdominal computerized tomography showing narrowing of the ileum (arrow) due to compression by a residual hematoma on the anal side of the anastomosis.

Contrast-enhanced CT revealed that the intra-abdominal hematoma was localized in the left and right lower abdomen, and percutaneous drainage was considered possible. On the 8th postoperative day, 18Fr drainage tubes were inserted into the left and right lower abdomen through CT-guided puncture, and a large amount of old blood was drained. After drainage, the inflammatory reaction and abdominal distension caused by the residual hematoma infection improved (CRP at 1.9 mg/dL and WBC of 5000/μL). No progression of anemia was observed. On the 14th postoperative day, drainage in the right lower abdomen changed from old blood to intestinal juice. A drain angiography revealed delayed anastomotic leakage at the ileostomy closure site (Fig. 2A). There were no signs of peritonitis with good drainage, but the drainage was approximately 100 to 200 mL, and flatus from the drain continued. No decrease in drainage was observed for the following 14 days.

## 5. Therapeutic intervention

**Figure 3.**
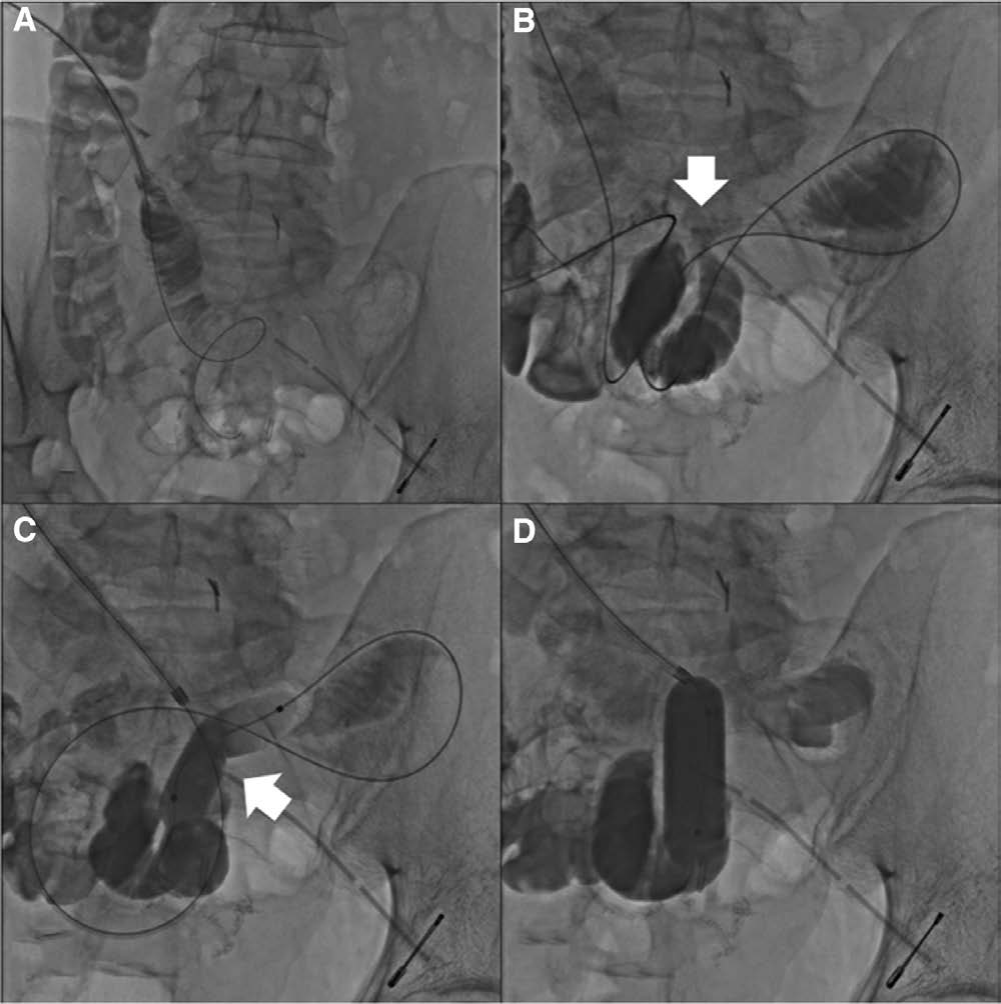
(A) Cordis BRITE TIP sheath introducer was placed at the fistula where anastomotic leakage occurred after the drain angiography. (B) Identification of the stenosis area of the ileum (arrow) due to the compression of the residual hematoma. (C) Balloon dilation of the stenosis area (arrow) using CRE PRO GI wireguided balloon. (D) Stenosis of the ileum improved after balloon dilation.

Although reoperation was considered, severe intra-abdominal adhesions were expected due to the history of past surgeries, postoperative bleeding, and anastomotic leakage. Therefore, reoperation was judged to be a high-risk procedure. Contrast-enhanced CT showed that an organized hematoma remained in the mesentery of the small intestine, and the ileum on the 10 cm distal side of the anastomosis was compressed near the remaining hematoma (Fig. 2B). Considering the stenosis of the ileum on the anal side of the anastomosis due to residual hematoma as the cause of anastomotic leakage, fluoroscopic balloon dilation of the stenosis area through the drain on the 24th postoperative day was attempted. A Cordis BRITE TIP® sheath introducer (8 Fr × 23 cm) was placed at the fistula where anastomotic leakage occurred after the drain angiography. A radifocus GT wire (0.016 inches, 180 cm, 90°) (Terumo, Tokyo, Japan) and a Renegade HI-FLO microcatheter (135 × 20 cm) (Boston Scientific, Tokyo, Japan) were used to reach the terminal ileum. Using Selecon MP catheter II (6Fr, 80 cm, φ20 mm) (Terumo), the stenosis area was identified in the ileum due to compression of the hematoma where the balloon could not pass (Fig. 3A and B).

Fluoroscopic balloon dilation was performed on the stenosis of the ileus using a CRE PRO GI wireguided balloon (180 cm, 15–18 mm) (Boston Scientific), inflating to 16.5 mm diameter at 4.5 atm for 3 minutes (Fig. 3C and D).

## 6. Follow-up and outcomes

From the day after the balloon dilatation, the amount of drainage decreased markedly, and flatus and defecation were observed from the anus. One week after balloon dilation, the drainage was almost zero, and the anastomotic leakage was judged to have resolved. The drain was gradually removed. Oral intake was good, and he had regular bowel movements. He was discharged on the 45th postoperative day. Since then, no recurrence of the stenosis or anastomotic leakage has been reported.

## 7. Discussion

We experienced a case of delayed anastomotic leakage after ileostomy closure caused by postoperative bleeding and compression of intraperitoneal hematoma. Prompt closure of the anastomotic leakage was achieved by fluoroscopic balloon dilatation of the ileum.

The complication rate of ileostomy closure is reported to be approximately 3% to 30%, and the perioperative mortality rate is reported to be 0% to 4%.^[5–7]^ The incidence of Clavien Dindo classification Grade III or higher complications has been reported to be 10.4%. Anastomotic leakage is known to be a severe complication with an incidence of approximately 5.1%.^[8]^ Anastomotic leakage after ileostomy closure is a challenging complication to treat, requiring reoperation to create a permanent ileostomy, or it can lead to an enterocutaneous fistula, leading to short bowel syndrome.^[9]^

However, postoperative bleeding after ileostomy closure is rare. It is known to be challenging to identify the source of postoperative bleeding and to stop the bleeding point even when the abdomen is reopened. If hemostasis cannot be achieved, abdominal compartment syndrome may occur, which can be a critical complication.^[10]^ Infection due to residual hematoma and persistent absorption fever also pose clinical problems.^[10]^ However, there are few reports of small bowel stenosis due to compression of residual hematoma as a complication of postoperative bleeding.

In this case, the cause of postoperative bleeding was unknown but was thought to be incomplete hemostasis in the omentum adhered around the stoma hole. Although it was possible to avoid the transition to abdominal compartment syndrome by reducing the hematoma volume using CT-guided drainage, complete drainage of the hematoma was complex, and the hematoma compressed the ileum on the anal side of the anastomosis. We believe that the increased pressure caused the delayed anastomotic leakage. Conservative treatment was complicated because the drainage volume could not be reduced, and reoperation was considered. However, because of the patient’s history of abdominal surgery, severe intra-abdominal adhesions due to postoperative bleeding and anastomotic leakage were expected. Therefore, the patient required a less invasive treatment.

In this case, the anal intestinal tract was reached through the fistula of the anastomotic leakage by performing the guidewire technique via drain angiography. This procedure enabled us to identify the stenotic area on the distal side of the anastomosis and perform the balloon dilation. Balloon dilation has been reported to help treat inflammatory bowel diseases, such as Crohn disease and benign small intestinal strictures.^[11]^ Although the proximal small intestine can be approached with enteroscopy, approaching the distal small intestine is often challenging. In this case, balloon dilation of the distal ileum was possible by placing an 8Fr sheath through the fistula of the anastomotic leakage and entering the distal ileum while protecting the fistula. Fluoroscopic balloon dilatation is a minimally invasive and effective procedure in cases of anastomotic leakage with stenosis on the anal side.

Restenosis is associated with balloon dilation in the small intestine.^[12]^ We believe that the early resumption of oral intake after the cause is resolved by balloon dilation and the bougie effect of stool passing through the dilation area could prevent restenosis.

## 8. Conclusions

In cases of anastomotic leakage that cannot be improved by drainage alone, it is essential to identify and treat the cause before choosing immediate reoperation. Furthermore, in cases of small intestinal anastomotic leakage after postoperative bleeding, compression of the intestinal tract due to a hematoma should be considered, and minimally invasive treatment of the cause should be attempted.

## Author contributions

**Conceptualization:** Teppei Kamada.

**Data curation:** Hironori Ohdaira, Takashi Aida, Daisuke Yamagishi, Junji Takahashi, Keigo Nakashima, Eisaku Ito, Norihiko Suzuki, Taigo Hata, Masashi Yoshida, Eigoro Yamanouchi.

**Writing – original draft:** Teppei Kamada.

**Writing – review & editing:** Hironori Ohdaira, Yutaka Suzuki.
